# Editorial: Expert opinions and perspectives in adoptive cell therapy for cancer: 2022

**DOI:** 10.3389/fimmu.2023.1257016

**Published:** 2023-07-20

**Authors:** Beatriz Martín-Antonio

**Affiliations:** Department of Experimental Hematology, Instituto de Investigación Sanitaria-Fundación Jiménez Diaz (IIS-FJD), University Autonomous of Madrid (UAM), Madrid, Spain

**Keywords:** CAR-T, CAR affinity, cytokine, gamma-delta T cells, tumor microenvironment

In the last decade, adoptive cell therapy (ACT) based on the isolation, genetic engineering and expansion of different types of immune cells is changing the treatment concept for cancer patients. ACT involves the infusion of tumor-infiltrating lymphocytes (TILs), engineered T cell receptor (TCR) cells, chimeric antigen receptor (CAR)-T cells, and natural killer (NK) cells. Specifically, clinical studies with CAR-T cells have made significant progress demonstrating superiority to the standard of care for patients with acute lymphoblastic leukemia and Non-Hodgkin Lymphoma (NHL), and establishing a paradigm shift by modifying the treatment of NHL patients (1, 2). Moreover, CAR-T cell therapy has achieved one more step becoming a tool to treat autoimmune diseases (3) and illustrating the potential of cellular immunotherapy to treat a high number of diseases.

Despite the success of CAR-T cell therapy in hematological malignancies, where there are six CAR-T cell products approved targeting either CD19 or BCMA (4), in solid tumors, this type of treatment has not achieved the expected success, and it is still in development. The main barriers found in solid tumors include: *1)* the lack of specific tumor antigens, that will cause on-target, off-tumor toxicity, *2)* the barriers of the tumor microenvironment that immune cells need to overcome and decrease the efficacy and persistence of CAR-T cells, *3)* the heterogeneity of cells in the tumor, which require several CARs targeting different antigens, *4)* and this treatment involves toxicities, known as cytokine release syndrome and “Immune effector cell-associated neurotoxicity syndrome”, that need to be carefully managed during patient treatment.

In addition, in hematological malignancies, still, there is a proportion of patients that might not receive their treatment due to a failure in CAR-T cell production, and a percentage of patients that achieve complete responses end up relapsing, mainly due to a loss of expression of the target antigen, and a lack of efficacy and persistence of CAR-T cells. In this regard, novel studies indicate the relevance of the CAR’s affinity towards the tumor antigen in the loss of target antigen expression and in the efficacy of the treatment. Moreover, modifications in the CAR construct and different small molecules added during the manufacturing of CAR-T cells aim to enhance the persistence of CAR- T cells. Furthermore, gene editing techniques to obtain allogeneic Universal CAR-T cells will allow to treat more patients.

In this Research Topic “*Expert Opinions and Perspectives in adoptive cell therapy for cancer: 2022*”, we provide a critical evaluation by leading experts in the state of ACT for cancer treatment. Experts offer their perspectives and analyze current challenges, the latest discoveries, and the future in the field.

First, Martinez-Cibrian et al. discuss their experience developing academic CAR-T cells targeting CD19, named ARI-0001 cells or Var-cel. ARI-0001 cells have been approved under the Hospital Exemption clause for the treatment of patients older than 25 years of age with relapsed/refractory acute lymphoblastic leukaemia, and have been granted PRIority MEdicines designation by the European Medicines Agency for the same indication. ARI-0001 cells have equivalent quality as those produced at industrial scale, in terms of manufacturing, and speed of response from patient identification to product delivery. The authors explain the steps and the hurdles found during the manufacturing process since apheresis to cell infusion (Vein-to-vein). ARI-0001 cells emerge as an initiative that could be replicated in other academic institutions, as valid alternatives to industrial CART-cells.

An essential aspect of CAR-T cell therapy’s success highlighted in this Research Topic is the CAR’s affinity towards the tumor antigen. This event is discussed in solid tumors by Mao et al., where they analyze 38 clinical trials of patients receiving CAR-T cells, concluding that CARs with moderate affinity have better efficacy than high and low-affinity CARs. Most antibodies approved for their use in patients are of high affinity, suggesting a critical aspect to deliberate.

The third paper of this series by Chen et al. reviews the state of CAR-T cells in thoracic malignancies, highlighting tumor antigens that are being targeted, and strategies to improve the homing of CAR-T cells to the tumor, and fight the TME. Moreover, to fight the TME and to enhance the persistence of CAR-T cells, one of the options is the development of “cytokine help intensified CAR” T cells (CHIC) reviewed by Thomas and Abken Indeed, the addition of cytokines or cytokine receptors in the CAR construct activates the activity of endogenous immune cells in the patient, enhances the efficacy of the treatment and importantly, avoids the need to add exogenously cytokines with their corresponding toxicity in the patients.

Zhang et al. discuss how different small molecules added during the production of CAR-T cells, achieve a product that is more “fit” and less immunosenescent, which will have enhanced persistence in the patients. These small molecules include protein kinase inhibitors, Bruton’s tyrosine kinase, and tyrosine kinase inhibitors, and immune modulators. Some of these products have already been tested in the clinic, demonstrating their potential. Moreover, they propose novel vasoactive intestinal peptide receptor antagonists (VIPR-ANT) peptides as emerging candidates to enhance cell-based immunotherapy by promoting a T-cell product that supports a less immunosuppressive environment.

The final paper in this series by Meringa et al. discusses using γδTCRs engineered T-cell therapies for treating solid tumors. γδTCRs offer an advantage compared to CAR- T cells as γδT cells supply T cells with natural TCR signaling, and their genetic modification improves co-stimulation, T cell fitness, and enhanced homing to the tumor tissue.

Let’s read the authors’ expert opinions to understand how this exciting field of research will evolve in the following years to achieve better and more permanent responses in the treatment of cancer patients.

## Author contributions

The author confirms being the sole contributor of this work and has approved it for publication.

## References

[1] Sharma P Kasamon YL Lin X Xu Z Theoret MR Purohit-Sheth T. FDA approval summary: axicabtagene ciloleucel for second-line treatment of large B-cell lymphoma. Clin Cancer Res (2023). doi: 10.1158/1078-0432.CCR-23-0568. [Epub ahead of print]. PMC10767767 37405396

[2] Westin JR Oluwole OO Kersten MJ Miklos DB Perales M-A Ghobadi A. Survival with axicabtagene ciloleucel in large B-cell lymphoma. N Engl J Med (2023). doi: 10.1056/NEJMoa2301665. [Epub ahead of print]. 37272527

[3] Mougiakakos D Krönke G Völkl S Kretschmann S Aigner M Kharboutli S. CD19-targeted CAR T cells in refractory systemic lupus erythematosus. N Engl J Med (2021) 385:567–9. doi: 10.1056/NEJMc2107725 34347960

[4] Chen Y-J Abila B Mostafa Kamel Y. CAR-T: what is next? Cancers (Basel) (2023) 15:663. doi: 10.3390/cancers15030663 36765623 PMC9913679

